# Patterns of treatment seeking behavior for mental illnesses in Southwest Ethiopia: a hospital based study

**DOI:** 10.1186/1471-244X-11-138

**Published:** 2011-08-22

**Authors:** Eshetu Girma, Markos Tesfaye

**Affiliations:** 1 Department of Health Education and Behavioral Sciences, Jimma University, Jimma, Ethiopia; 2 Department of Psychiatry, Jimma University, Jimma, Ethiopia

**Keywords:** 'mental illness', 'treatment seeking', 'pathways to care', 'Ethiopia'

## Abstract

**Background:**

Early recognition of the signs and symptoms of mental health disorders is important because early intervention is critical to restoring the mental as well as the physical and the social health of an individual. This study sought to investigate patterns of treatment seeking behavior and associated factors for mental illness.

**Methods:**

A quantitative, institution-based cross sectional study was conducted among 384 psychiatric patients at Jimma University Specialized Hospital (JUSH) located in Jimma, Ethiopia from March to April 2010. Data was collected using a pretested WHO encounter format by trained psychiatric nurses. Data was analyzed using SPSS V.16.

**Result:**

Major depression disorder 186 (48.4%), schizophrenia 55 (14.3%) and other psychotic disorders 47 (12.2%) were the most common diagnoses given to the respondents. The median duration of symptoms of mental illness before contact to modern mental health service was 52.1 weeks. The main sources of information for the help sought by the patients were found to be family 126 (32.8%) and other patients 75 (19.5%). Over a third of the patients 135 (35.2%), came directly to JUSH. Half of the patients sought traditional treatment from either a religious healer 116 (30.2%) or an herbalist 77 (20.1%) before they came to the hospital. The most common explanations given for the cause of the mental illness were spiritual possession 198 (51.6%) and evil eye 61 (15.9%), whereas 73 (19.0%) of the respondents said they did not know the cause of mental illnesses. Nearly all of the respondents 379 (98.7%) believed that mental illness can be cured with modern treatment. Individuals who presented with abdominal pain and headache were more likely to seek care earlier. Being in the age group 31-40 years had significant statistical association with delayed treatment seeking behavior.

**Conclusions:**

There is significant delay in modern psychiatric treatment seeking in the majority of the cases. Traditional healers were the first place where help was sought for mental illness in this population. Most of the respondents claimed that mental illnesses were caused by supernatural factors. In contrast to their thoughts about the causes of mental illnesses however, most of the respondents believed that mental illnesses could be cured with biomedical treatment. Interventions targeted at improving public awareness about the causes and treatment of mental illness could reduce the delay in treatment seeking and improve treatment outcomes.

## Background

Mental health is one of the vital components of health [1,2]. There is evidence that mental and physical illnesses may accompany, follow, or precede one another. There is also evidence which indicated that mental disorders increase the risk of physical illness and vice versa [3]. Persons with mental illness receive a wide range of responses across cultures. In the developing world, they are subjected to severe stigma and mistreatment, but in some cases are helped by community support structures. Traditional beliefs that attribute psychiatric disorders to moral transgression and misconstrue the dangerousness of patients lead to feelings of shame and fear of persons with mental illness. Such community values and beliefs influence treatment seeking behavior, treatment outcomes, and even determine the way mental health is practiced [4,5]. Beliefs regarding the causes of mental illnesses hover between the natural and the supernatural. They vary according to an individual's level of education and socioeconomic class. In less educated areas of the countryside, there exist a number of supernatural explanations of mental illness which include possession by spirit, black magic, or astrological misalignment [4,6,7]. Over 450 million people are estimated to be suffering from mental disorders in the world today. Only a small proportion of these people receive any form of modern treatment, and most untreated cases are found in low income countries [8]. In Ethiopia, for example, less than 10% of persons with severe mental illness had contact with modern psychiatric services. Fifteen to twenty percent of people who attend general medical clinics do so because of mental disorders, although their mental health problems are often not recognized [9,10]. Modern psychiatric services are very scarce, inaccessible, and relatively expensive for the majority of the population in Ethiopia. Therefore, patients usually resort to modern mental health-care services only after they have failed to recover after receiving traditional treatments. It is also a common practice in Ethiopia for family members to care for and support persons with mental illness at home. A study also showed that less than half of mental illness patients directly contacted a mental hospital, and the median delay between onset of illness and arrival at the psychiatric hospital was 38 weeks [11]. In pluralistic medical settings, laypeople choose what to do first, second, third, and fourth from a variety of treatment options [11,12]. A systematic analysis of the sequence of treatment options sought provides insights into patients' patterns of resort and suggests a tentative theory for how laypeople make medical choices. The strength of pathway models is that they depict health seeking as a dynamic process. Factors are sequentially organized, according to the different key steps (i.e. recognition of symptoms, decision making, medical encounter, evaluation of outcomes, and re-interpretation of illness) which determine the course of the therapy path [13,14]. Hence this study has investigated the patterns of health seeking behavior for mental illnesses in JUSH.

## Methods

We conducted a quantitative, hospital-based cross sectional study to explore the patterns of health seeking behavior and related factors for mental illness from March to April, 2010, at JUSH, Ethiopia. The hospital is located in the city of Jimma, a town in southwestern Ethiopia 345 km from the capital city of Addis Ababa. The psychiatric facility at the hospital is staffed by one psychiatrist and three psychiatric nurses, and it has 26 inpatient beds. We approached 384 consecutive new patients attending the psychiatric facility of JUSH during the study period. The sample size was determined with a single population proportion formula by assuming that 50% of the patients will come early for mental illness treatment at a psychiatric facility (to obtain maximum sample size) with 95% confidence interval. Consecutive patients attending outpatient department of psychiatry with a new episode of illness during the study period were included in the study. Caregivers were interviewed whenever the person with mental illness could not respond due to the illness, had expression and/or hearing problems, or whenever the patient was younger than eighteen years. Patients were enrolled until the required sample size was obtained. Registration records were reviewed each day to select study subjects who were eligible for the study. Data was collected using a pretested questionnaire which was administered using the face-to-face interview method. Information regarding psychiatric diagnoses of participants was obtained from their medical card. Data was collected by trained psychiatric nurses who were fluent in Afaan Oromo and Amharic languages (local languages). The questionnaire was adopted from World health organization encounter form for Pathways to care [15] and items that assess the perception of patients on mental illness was developed from the Good's pathway model [14]. For the purpose of this study, a person with mental illness was defined as any patient who received any psychiatric diagnosis after being evaluated by mental health professionals. Mental health remedies sought for mental illness were categorized as religious (rituals/practices, herbalists and other traditional healings), biomedical (government and private 'modern' health institutions), and self care (home remedies). Perceived causes of mental illnesses were assessed based on the perception of the respondents and were categorized as a "traditional explanation" (i.e. spirit possession) or "modern explanation" (i.e. pathogens) Perceived susceptibility to mental illness was assessed by asking the open question 'who do you think mental illness affects?' Perceived severity was measured by asking 'how do you rate the severity of mental illnesses generally?' with possible responses being 'highly severe,' 'moderately severe,' 'less severe,' and 'not at all severe'. To assess the belief of remedies for mental illness, subjects were asked, 'can mental illnesses be cured?' To categorize significant delay for seeking mental health treatment, we took 38 weeks as a reference point from a study conducted at Amanuel mental specialized hospital in Addis Ababa, Ethiopia [11]. Hence we categorized individuals who sought mental health treatment from the psychiatric hospital above 38 weeks as delayed and below 38 weeks as not delayed. Data was checked for completeness and consistency and analyzed using SPSS16 statistical software. Descriptive statistics were presented using summary tables and graphs. Cross-tabulations and multivariate logistic regression was done to identify the most important predictor variables of mental health treatment seeking behavior. Ethical approval was obtained from ethical review board of Jimma University. Written informed consent was obtained from the participants. For literate people, they themselves read the already prepared consent. But for those who could not read, the data collectors read for them and obtained their signature or finger print to affirm their consent.

## Result

### Characteristics of the study participants

The majority of the respondents were male 238 (62.0%). Individuals less than 20 years old and those between 21-30 years accounted for the largest proportion of subjects (111 (28.0%) and 173 (45.1%) respectively). The mean (SD) age of the patients was 28.75 (-0.12) years with maximum of 80 years and minimum of 10 years. More than half of the study population was unmarried 215 (56.0%). Nearly three quarters 285 (74.2%) of the participants had attended formal education. Muslim is the major religious group 246 (64.1%) followed by Ethiopian Orthodox 84 (21.9%). Student and farmer were the most common occupations described accounting for 100 (26.0%) and 93 (24.2%) respectively. The mean family (SD) monthly income was 510.3 (484.1) Ethiopian birr (1 USD = 17 ETB). The majority of the study population 338 (88.0%) was from Jimma zone where JUSH is located. Those coming from rural regions accounted for 204 (53.1%) of the total sample (Table 1). One hundered and thirteen subjects (29.4%) had no caregiver during the time of interview.

**Table 1:** socio-demographic distribution of mental illness patients at JUSH psychiatry department, Ethiopia, 2010

Variable	Not delayed (total = 134)	Delayed (total = 250)	Total (%) (N = 384)
**Gender**			
**Male**	88(22.9)	150(39.1)	238 (62.0)
**Female**	46(12.0)	100(26.0)	146 (38.0)
**Age**			
**< 21**	53(13.8)	58(15.1)	111 (28.9)
**21-30**	56(14.6)	107(27.9)	163 (42.4)
**31-40**	15(3.9)	48(12.5)	63 (16.4)
**41-50**	6(1.6)	18(4.7)	24 (6.2)
**51-60**	1(0.3)	12(3.1)	13 (3.4)
**> 60**	3(0.8)	7(1.8)	10 (2.6)
**Marital status**			
**Single**	88(22.9)	127(33.1)	215 (56.0)
**Married live together**	38(9.9)	88(22.9)	126 (32.8)
**Married not live together**	3(0.8)	7(1.8)	10 (2.6)
**Divorced**	3(0.8)	20(5.2)	23 (6.0)
**Widowed**	2(0.5)	8(2.1)	10 (2.6)
**Educational status**			
**Attended formal education**	107(27.9)	178(46.4)	285 (74.2)
**Cannot read and write**	24(6.2)	58(15.1)	82 (21.4)
**Read and write only**	3(0.8)	14(3.6)	17 (4.4)
**Religion**			
**Muslim**	85(22.1)	161(41.9)	246 (64.1)
**Orthodox**	29(7.6)	55(14.3)	84 (21.9)
**Protestant**	20(5.2)	33(8.6)	53 (13.8)
Others*	0(0.0)	1(0.3)	1 (0.3)
**Ethnicity**			
**Oromo**	91(23.7)	165(43.0)	256 (66.7)
**Amhara**	13(3.4)	39(10.2)	52 (13.5)
**Dawro**	9(2.3)	8(2.1)	17 (4.4)
**Keffa**	7(1.8)	8(2.1)	15 (3.9)
**Yem**	3(0.8)	6(1.6)	9 (2.3)
Others**	11(2.9)	24(6.2)	35 (9.1)
**Occupation**			
**Student**	48(12.5)	52(13.5)	100 (26.0)
**Farmer**	27(7.0)	66(17.2)	93 (24.2)
**House wife**	17(4.4)	41(10.7)	58 (15.1)
**Government employee**	17(4.4)	28(7.3)	45 (11.7)
**Merchant**	8(2.1)	21(5.5)	29 (7.6)
**Daily laborer**	4(1.0)	8(2.1)	12 (3.1)
Others***	13(3.4)	34(8.9)	47 (12.2)
**Place of origin**			
**In Jimma zone**	119(31.0)	219(57.0)	338 (88.0)
**Outside of Jimma zone**	15(3.9)	31(8.1)	46 (12.0)
**Type of residency**			
**Rural**	65(16.9)	115(29.9)	204 (53.1)
**Urban**	69(18.0)	135(35.2)	180 (46.9)

*Catholic, traditional religions **Siltie, Gurage, Tigre, Wolayta, Hadya

*** Taxi driver, pension, and religious leader

### Mental health seeking behavior

The respondents came to JUSH for treatment of mental illness after a mean of 231.6 weeks and median of 52.1 weeks (maximum of 38 years and minimum of 26.4 hours) from the onset of symptoms of mental illness. Before visiting to JUSH psychiatric facility, 51 (13.3%) had used medication for mental health from biomedical institutions with or without prescription. Considering all forms of transportation, the median time to reach to JUSH psychiatric facility was 1 hour with maximum of 23.5 hours and minimum of 1.0 minutes. Only 63 (16.4%) subjects had previously visited JUSH for mental illness treatment. The majority of the respondents 145 (37.8%) reported that they found their preferred sites of treatment on their own without being informed by others. Family members 126 (32.8%) and former patients 75 (19.5%) were the most common external sources of information for seeking treatment (Table 2). Most of the respondents 250 (65.1%) came to treatment after significant delay from the onset of their symptoms.

**Table 2:** Reinforcing factors for seeking mental health care at JUSH psychiatry department, Ethiopia, 2010

Variable	Frequency	Percent
**who insisted/informed to visit (N = 384)**		
No one	145	37.8
Family	126	32.8
Former patient	75	19.5
Friend	24	6.2
Previous provider	22	5.7
Relative	14	3.6
Neighbor	18	4.7
Religious leader	1	0.3
Other people	4	1.0

Total > 384 due to multiple response

**Figure 1:**
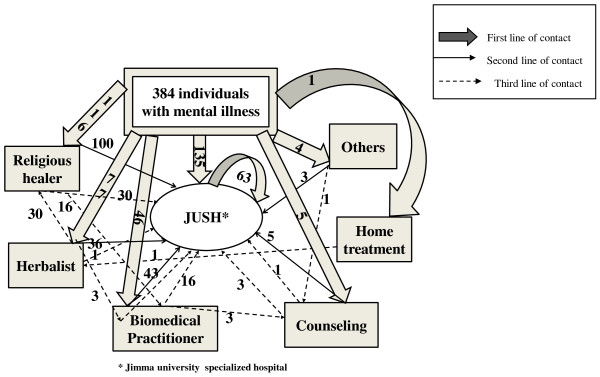
**patterns of resort for mental illness patients at JUSH psychiatry department, Ethiopia, 2010**.

One hundred thirty five (35.2%) of the study participants came directly to JUSH. More than half of the patients sought traditional treatment from either a religious healer 116 (30.2%) or an herbalist 77 (20.1%) before they came to the hospital. The majority of patients attended JUSH as their second or third order treatment location (Figure 1).

### Perception of mental illnesses

Eighty four (21.9%) subjects had at least one family member with mental illness. Among these family members, the majority (44.1%) of them were siblings of the respondents. The leading psychological and behavioral problems/complaints mentioned were anxiety-related 365 (95.1%), depression-related 360 (93.8%) and other neurotic symptoms 296 (77.1%). The most commonly mentioned somatic symptoms were sleep disturbance 348 (90.6%), headache 222 (57.8%) and weakness/lethargy 123 (32.0%) (Table 3). The majority of the study population believed that vulnerable groups for mental illness include people who are angry and stressed 145 (37.8%), who use drugs 100 (26.0%), and people with crisis 66 (17.2%). The most common explanations given on the causation of mental illness were spiritual possession 198 (51.6%), and evil eye 61 (15.9%) where as 73 (19.0%) of the respondents said they did not know the cause of mental illnesses. Nearly all of the respondents 379 (98.7%) believed that mental illness can be cured with modern treatment. The majority of the subjects 376 (97.9%) believe mental illness is a severe health problem. Only 61 (15.9%) reported that the community perceives mental illness as not as such shameful and not at all shameful (Table 4).

**Table 3:** Perceived sign and symptoms for mental health problems of patients at JUSH psychiatry department, Ethiopia, 2010

Perceived Illness	No (N = 384)	%
**Psychological and Behavioral Problems**		
Anxiety related	365	95.1
Depression related	360	93.8
Interpersonal problems	318	82.8
Other neurotic symptoms	296	77.1
Violent or aggressive behavior	254	66.1
Other disturbed behavior	201	52.3
Suicide attempt	181	47.1
Drug related problems	147	38.3
Alcohol related problem	79	20.6
Other organic symptoms	61	15.9
Fits/alterations of consciousness	34	8.9
Psychotic symptoms	16	4.2
**Somatic symptoms**		
Sleep disturbance	348	90.6
Headache	222	57.8
Weakness/lethargy	123	32.0
Loss of weight	102	26.6
Dizziness	87	22.7
Fever	56	14.6
Abdominal pain	50	13.0
Cough/cold/influenza	36	9.4
Back/chest pain	34	8.9
Genito-urinary symptoms	32	8.3
Other somatic symptoms	16	4.2

**Table 4:** perception of respondents on mental illness at JUSH psychiatry department, Ethiopia, 2010

Characteristics	Frequency	Percent
**What kind of people mental illness affects**		
Angry and stressed	145	37.8
people who use drug	100	26.0
People with crisis	66	17.2
Those who think a lot	50	13.0
Others*	23	6.0
**Perceived Causes of mental illness**		
Spiritual possession	198	51.6
I do not know	73	19.0
Evil eye	61	15.9
Family history	57	14.8
Sinful act	41	10.7
Pathogens	37	9.6
Stress	13	3.4
Others	23	6.0
**Believe mental illness can be cured**		
Yes	379	98.7
I am not sure	4	1.0
No	1	0.3
**Perceived severity of Mental illness**		
Very high severe	119	31.0
high severe	199	51.8
Severe	58	15.1
less severe	8	2.1
**Community perception for mental illness**		
Very high shameful	51	13.3
Highly shameful	152	39.6
Shameful	120	31.2
Not as such shameful	28	7.3
Not at all shameful	33	8.6

others: accident, addiction, curse, God's will, fright, crisis, love, over wish, younger

Others * khat (natural stimulant from Catha Edulis plant) abuse, cigarette, alcohol abuse

### Types of medically diagnosed mental illness

**Figure 2:**
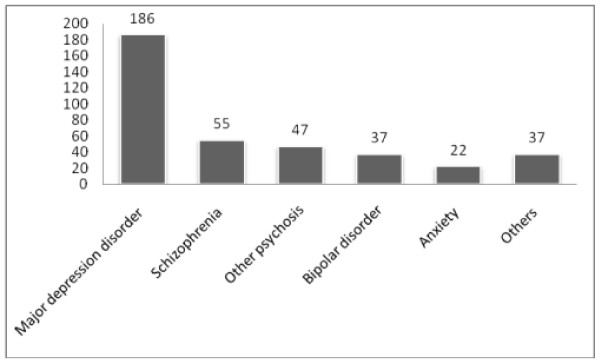
**medically diagnosed mental health problems among mental illness patients at JUSH psychiatry department, Ethiopia, 2010**.

The medical diagnosis of the study participants indicated that major depressive disorder 186 (48.4%), schizophrenia 55 (14.3%) and other psychosis 47 (12.2%) were the leading kinds of mental health problems (Figure 2).

### Determinants of treatment seeking for mental health

In the bivariate analysis, age, marital status, presence of other family member with mental illness, the type of diagnosed mental illness, and source of information about mental health service had significant statistical association with early treatment seeking behavior (p < 0.05). Having other neurotic symptoms, interpersonal problems, suicide attempt, headache, abdominal pain and fever also had significant statistical association with early treatment seeking for mental illness (p < 0.05).

Results from the multivariate analysis showed that individuals whose age was 31-40 were 10.7 times more likely to seek treatment later than subjects under 20 years of age [OR-10.7, 95%CI (1.99, 56.99)]. Individuals who had symptoms of abdominal pain [OR-6.1, 95%CI (1.32, 28.56)] and headache [OR-3.3, 95%CI (1.17, 9.24)] were more likely to seek care early than without these signs and symptoms. But people with a history of suicide attempt were less likely to seek treatment early than without an attempt [OR-0.2, 95%CI (0.09, 0.65)] (Table-5).

**Table 5:** predictors of mental health seeking behavior among psychiatric patients at JUSH psychiatry department, Ethiopia, 2010

Variable	Early mental health seeking behavior		OR (95.0% C.I.)
	Yes	NO	
**Suicide attempt**			
No	82(40.4%)	121(59.6%)	1
Yes	52(28.7%)	129(71.3%)	0.2(0.09,0.65)
**Headache**			
No	43(26.5%)	119(73.5%)	1
Yes	91(41.0%)	131(59.0%)	3.3(1.17, 9.24)
**Abdominal pain**			
No	109(32.6%)	225(67.4%)	1
Yes	25(50.0%)	25(50.0%)	6.1(1.32,28.56)
**Patient age (in years)**			
< 21	53(47.7%)	58 (52.3%)	1
21-30	56(34.0%)	107 (65.6%)	0.9(0.28,2. 82)
31-40	15(23.8)	48(76.2%)	10.7(1.99,56.99)
41-50	6 (25.0%)	18(75.0%)	1.1(0.12,10.69)
51-60	1(7.7%)	12(92.3%)	5.3(0.27,106.61)
> 60	3(30.0%)	7(70.0%)	2.5(0.10,62.67)

## Discussion

Generally, most of the respondents 250 (65.1%) contacted a modern psychiatric treatment facility after significant delay from the onset of their symptoms. The median time of delay with this study was higher (52.1 weeks) than a study conducted in Addis Ababa, Ethiopia at Amanuel mental specialized hospital (38 weeks) [11]. It was also extremely high in comparison with a study conducted in Eastern Europe in which the median time was only 3 weeks [6]. Around 35.2% of the study subjects contacted JUSH as the first place of care, but the remaining subjects have visited either other biomedical or traditional care. In comparison to the study from Addis Ababa where 41% sought treatment directly from the mental hospital [11], less patients came directly to JUSH for treatment. One possible explanation for this discrepancy is that the majority of the subjects in this study was from rural areas and was faced with much longer geographic distances from the psychiatric facility than those in the study conducted in Addis Ababa. These larger distances may have increased the likelihood to contacting traditional healers before seeking treatment at JUSH.

A significant proportion of the study subjects (52.3%) were suggested to seek care at JUSH by either family or former patients. Similarly, a finding from Eastern Europe showed that the suggestion to seek care most often came from family or friends [6]. This might reflect the general lack of awareness on mental illness and the availability of mental health care among Ethiopians. The most common types of medically diagnosed mental illnesses were major depression, schizophrenia and other psychosis respectively. The most prevalent diagnosis in the Eastern Europe study was mood and neurotic disorders followed by schizophrenia [6].

A number of people did not know the causes of mental illness and most said that the perceived causes of mental illnesses were supernatural power and evil eye. A community based study conducted in Agaro town which is around 50 Kms away from Jimma town showed that poverty was the most commonly perceived cause of mental health problems followed by 'God's will' [16]. The finding that 98.7% of the respondents believed that mental illnesses are curable is alarming because it might reflect the lack of awareness regarding the chronic course of mental illnesses--particularly those more severe in nature. This could imply that there is an unrealistic expectation from whatever help is sought, and there is a risk of consequent dissatisfaction with the outcome and which may perhaps lead to poor adherence to treatment. On the other hand, the social desirability bias might have contributed to such a high figure since the study was conducted in a psychiatric facility and the data collectors were psychiatric nurses. The paradoxical finding that most of our study participants believed that spiritual factors caused the mental illness and that modern medications helped to cure the illness suggest that despite their belief in supernatural causes, their treatment seeking behavior was pragmatic.

Most of the respondents perceived that mental illnesses generally are severe health problems. Their perception was similar to another general community based study which found that Epilepsy was considered as the most serious problem followed by schizophrenia [16]. Most of the study participants perceive that mental illnesses are considered as a shame in the community. This perception about the community attitude towards mental illnesses contrasts with a study on community attitude towards mental illnesses where more than forty percent had positive attitude towards living with persons with mental illnesses [16]. This might be because of felt stigma by the person with mental illness. Such negative feelings as shame and guilt might contribute to delayed treatment seeking for mental illness. Unlike that of the study conducted in Addis Ababa [11], our study demonstrated that age of the patient had significant statistical association with early treatment seeking behavior. Somatic problems were also predictors of early treatment seeking behavior for mental illness. Persons with somatic symptoms may present to the primary care early in the course and may then get referred for psychiatric assessment. The reason for the association between having attempted suicide and delayed treatment seeking is unclear from our study. It is possible that people who are depressed might have not sought treatment for a long time which led to a worsening of their symptoms which rendered them too depressed to attempt suicide. Another explanation could be that the majority of respondents being either Muslim or Coptic Christians which stigmatize suicide and hence people might be hesitant to show up. Nevertheless, this needs further investigation.

Our study suffers social desirability bias as the setting is a psychiatric facility and the data collectors were staff members of the hospital. There might be recall bias on the onset of the mental illness and settings for treatment which were sought. It may not be generalizable to community as only a small proportion of persons with mental illness present to modern psychiatric treatment.

## Conclusion

Most of the respondents came to treatment after significant delay. Only 35% of the patients with mental illness came directly to modern psychiatric treatment. There was a paradox between their belief of the causes of mental illnesses and the type of treatment sector, as a large proportion of subjects felt that mental illness was caused by supernatural means but was curable by biomedical treatment. Individuals who had the sign and symptoms of abdominal pain and headache were more likely to seek care early. Being within the age of 31-40 years was associated with seeking psychiatric help much earlier than other age groups. Interventions targeted at improving public awareness about the causes and treatment of mental illness could reduce delay in treatment and thus improve treatment outcomes.

## Competing interests

The authors declare that they have no competing interests.

## Authors' contributions

EG designed the study, participated in the data collection, analyzed the data and drafted the manuscript. MT was involved in the design, analysis and reviewed the article critically.

All authors read and approved the final manuscript.

## Pre-publication history

The pre-publication history for this paper can be accessed here:

http://www.biomedcentral.com/1471-244X/11/138/prepub
